# A Literature Review of the Pharmacological Effects of Jujube

**DOI:** 10.3390/foods13020193

**Published:** 2024-01-06

**Authors:** Deqi Zhu, Ning Jiang, Ning Wang, Yufen Zhao, Xinmin Liu

**Affiliations:** 1 Institute of Drug Discovery Technology, Ningbo University, Ningbo 315211, Chinawangning2@nbu.edu.cn (N.W.); zhaoyufen@nbu.edu.cn (Y.Z.); 2 Research Center for Pharmacology and Toxicology, Institute of Medicinal Plant Development (IMPLAD), Chinese Academy of Medical Sciences and Peking Union Medical College, Beijing 100193, China; jiangning0603@163.com

**Keywords:** jujube, pharmacological effect, active ingredient

## Abstract

Jujube is a plant native to China that could be used in medicine and food. Its dried fruit is a superior herb commonly used in traditional Chinese medicine formulations for its calming effect and for nourishing the blood and strengthening the spleen and stomach. Jujube contains numerous active components including polysaccharides, phenols, and triterpene acids, which show a diverse array of pharmacological activities such as neuroprotection and the prevention and treatment of cardiovascular diseases. In this paper, the research status of jujube over the past two decades has been statistically evaluated. Meanwhile, by tracking the latest research advances, the pharmacological efficacy and molecular mechanisms of jujube are exhaustively expounded to provide specific and systematic references for further research on the pharmacological effects of jujube and its application in the food and pharmaceutical industries.

## 1. Introduction

*Ziziphus jujuba* Mill, also known as jujube or Chinese date, is a species belonging to the Rhamnaceae family and has evolved from the asexual reproduction of *Z. jujuba* var. *spinosa* [1]. Based on maturity, jujube can be broadly classified as immature fresh and mature dried jujubes. Fresh jujube is a delicious and nutritious fruit consumed worldwide, while dried jujube is made into fruit snacks or brewed with tea, and it is also commonly used in traditional Chinese medicine (TCM) formulations for both medicinal and flavoring purposes.

Jujube has been used as a Chinese herb for more than 2000 years and applied in healthcare as a dietary supplement and therapy, owing to its high nutritional and medicinal value. According to the ancient Chinese medicine book Treatise on Typhoid and Miscellaneous Diseases, jujube is often used in TCM formulations to strengthen the spleen and stomach and harmonize the body and the mind [2]. The Yellow Emperor’s Classic of Internal Medicine and Shennong’s Herbal have listed jujube as a superior herb for calming the mind, improving sleep, and nourishing the blood [3,4]. With the flourishment of the jujube industry during the past three decades, the medicinal value of jujube has gained an escalating focus, and its potential pharmacological effects and mechanisms have been continuously revealed. This paper is a systematic review of the pharmacological effects of jujube and the potential active ingredients and mechanisms. We hope to provide a reference for further exploring the medicinal value of jujube and promoting its application in the food and pharmaceutical industries.

## 2. Data Collection

**Figure 1:**
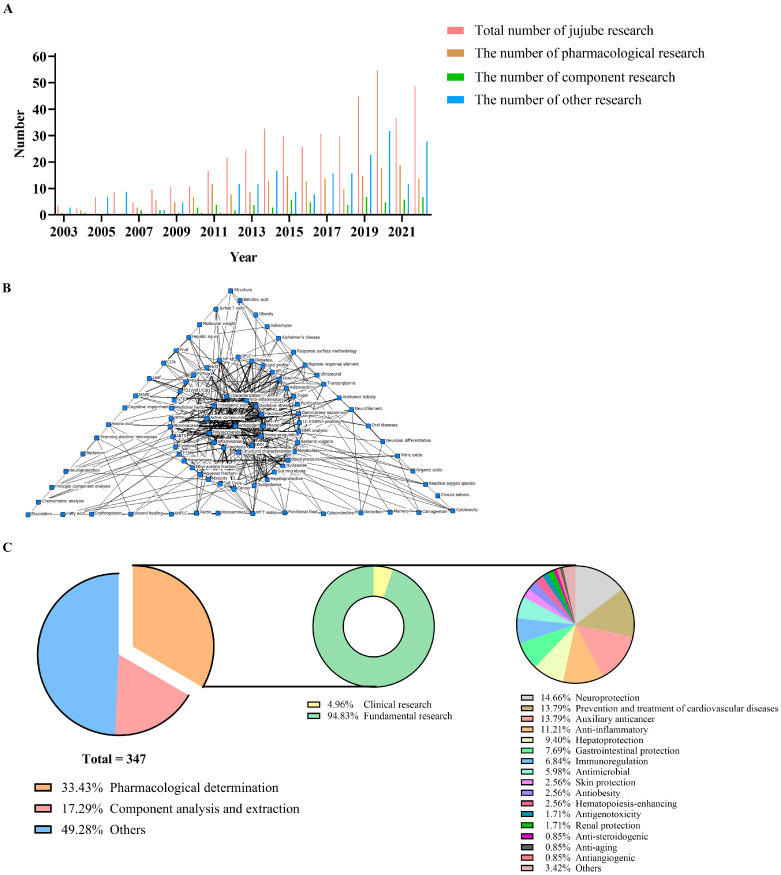
The growth, distribution and co-occurrence analysis of jujube research from 2003 to 2022. (**A**) Growth of jujube research; (**B**) Co-occurrence analysis of article keywords; (**C**) Distribution of jujube research.

PubMed was preliminary searched by typing (jujube[MeSH Major Topic]) AND (jujuba[Title/Abstract] OR jujube[Title/Abstract] NOT spinosa[Title/Abstract] NOT sour[Title/Abstract] NOT wild[Title/Abstract] NOT mauritiana[Title/Abstract]) with the year set from 2003 to 2022, and the result returned 400 papers. Subsequently, through assessing in detail, comments, reviews, and irrelevant articles were eliminated, and then 347 articles were screened out, covering pharmacological effects (33.43%), component analysis and extraction (17.29%), and other research including cultivation breeding, biological control and genome identification of jujube (49.28%). Figure 1 shows the growth and distribution of jujube research in the past twenty years, as well as the co-occurrence analysis of the article keywords. As can be seen from the figure, the related research on jujube has greatly increased since 2011 and has maintained a high growth rate since then (Figure 1A). The pharmacological mechanism of jujube is closely related to immunity, inflammation, and oxidation, and polysaccharides, triterpenic acids, and phenols are the main active components (Figure 1B). In the pharmacological research, most are preclinical studies on cells and animals, and only six cases are clinical studies. They have proven the diverse pharmacological effects of jujube, among which the most widely studied are neuroprotection and the prevention and treatment of cardiovascular diseases, as well as auxiliary anticancer, anti-inflammatory, hepatoprotection, and other effects. In addition, despite the scarcity of related studies, effects like anti-angiogenesis and anti-aging have also been reported (Figure 1C). Through a thorough study of these pharmacological papers, the active components and effects of jujube, as well as the potential mechanisms, were summarized.

## 3. Active Ingredients and Pharmacological Effects

**Figure 2:**
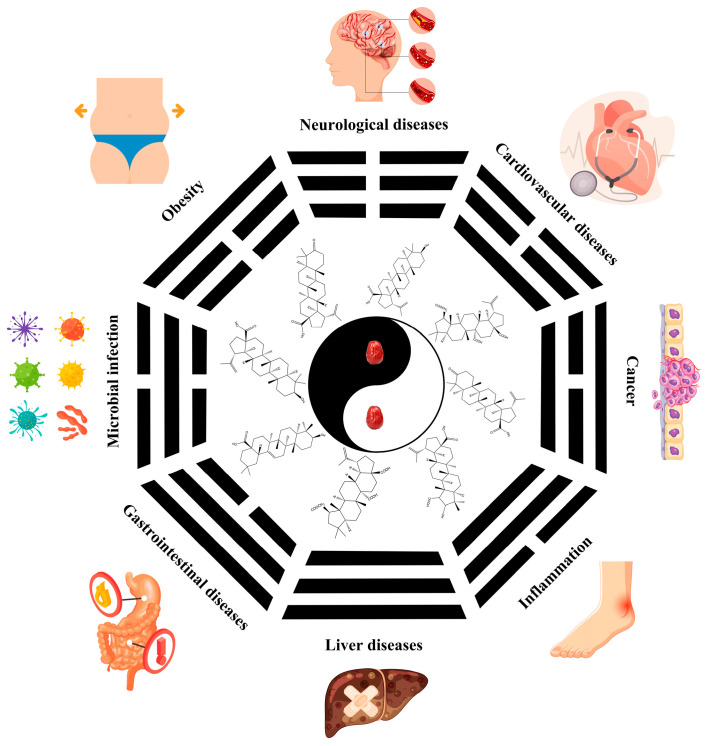
The pharmacological effects and the potential active ingredients of jujube.

Jujube contains a variety of nutrients and active substances, including polyphenols, polysaccharides, nucleotides, ascorbic acid, and triterpenoid acids [5]. Among them, ingredients such as polysaccharides and triterpenoid acids extracted from jujube have been proven to have diverse pharmacological activities, and the representative compounds are shown in Table 1. Polysaccharides are the most abundant and important, with high biological activity, low toxicity, and diverse pharmacological effects, including antitumor [6], hypolipidemic [7], and hepatoprotective [8]. Triterpenic acids contribute to the hepatoprotection, anti-inflammatory [9], and auxiliary anticancer [10] activity of jujube. Phenolic compounds exert strong antioxidant effects against free radicals and reactive oxygen species (ROS), and they exhibit skin protection effects as well as potential therapeutic activity against liver injury [11,12]. The alkaloids in jujube have been found to exert good antioxidant [13] and antiviral [14] activities. Moreover, the oleamide extracted from jujube exhibits choline acetyltransferase (ChAT)-activating and neurotoxic inhibitory effects [15], and a novel peptide snakin-z extracted from jujube was found to have antibacterial, antioxidant and cholinesterase inhibitory activities [16,17]. The pharmacological effects and the potential active ingredients of jujube are shown in Table 2. and Figure 2. The original document of the compound structural formulas is in File S1.

In this section, the pharmacological effects of jujube along with the potential active components and biological mechanisms are expounded using diseases or clinical symptoms as the classification criteria.

### 3.1. Neuroprotection

The therapeutic potential of jujube for neurological diseases is shown in Table 2. The jujube extract exerted neuroprotective effects in animal cerebral ischemia models by attenuating oxidative stress in different brain regions. A gavage of the jujube extract to cerebral ischemia gerbils upregulated SOD1 expression in the hippocampal CA1 region and attenuated lipid peroxidation, thereby protecting neurons from ischemic injury [29]. Similarly, reduced MDA and NO levels and increased SOD levels were found in the cerebral cortex, striatum, and hippocampus of rats with focal cerebral ischemia after the gavage of the jujube extract [30]. Alzheimer’s disease (AD) is an irreversible degenerative disease of the central nervous system characterized by the progressive degeneration of cognitive and memory functions. The possible causative mechanisms involve oxidative stress, defect of the cholinergic system, and neuroinflammation [31]. Promoted proliferation of hippocampal dentate gyrus neuronal cells and neuroblast differentiation and enhanced synaptic plasticity are potential mechanisms of jujube for improving learning and memory [32]. Additionally, jujube exerts protective effects on learning memory by reducing inflammation and oxidative stress, regulating cholinergic transport, as well as modulating apoptosis. In rat models of learning memory impairment, the levels of oxidative markers and pro-inflammatory factors, as well as the AChE activity, were significantly downregulated after treatment with jujube extract. Meanwhile, a significant improvement in behavioral parameters was observed in a Morris water maze [33]. Epilepsy is a common chronic neurological disorder manifesting as recurrent and irregular seizures and is often accompanied by the occurrence of cognitive disorders or impairment. Jujube is involved in epilepsy treatment by alleviating oxidative stress and regulating cholinesterase synthesis and release. As an illustration, decreased GSH levels as well as AChE and BChE activities in the rat brain tissue were reversed by jujube. Simultaneously, prolonged myoclonic latency and improved cognitive function were transparently observed [34]. Furthermore, a study has revealed that the protective effect of the jujube extract on epileptic rats was double that of the subtherapeutic doses of antiepileptic drugs alone [35].

**Table 2:** Therapeutic effects and potential pathways of jujube in the prevention and treatment for neurological diseases.

Diseases	Models	Type	Administration	Effects	Refs.
Cerebral ischemia	Rats and gerbils: I/R, MACo	In vivo	i.g.: 100 mg/kg 100, 250, 500 mg/kg	↑SOD ↑BDNF, NeuN-immunoreactive neurons ↓Reactive gliosis ↓HNE, MDA, NO ↓Neurological deficit score, motor dysfunction, cerebral infarct volume	[29,30]
AD	Rats and mice: scopolamine, D-galactose, NBM	In vivo	p.o.: 16, 32 mg/d (oleamide) ^1^ 14–16 mg/d 29, 57, 114 mg/kg	↑Learning and memory ↑SOD, FRAP, GSH ↑ACh ↑Neurons ↓ALT, AST, AChE, BChE, GFAP, Iba-1 ↓Caspase3, 9 ↓IL-1β, TNF-α, IL-6, INF-β	[15,31,33]
Epilepsy	Rats: maximal electroshock, pentylenetetrazole	In vivo	i.p.: 100, 250, 500 mg/kg p.o.: 500 mg/kg	↑Learning memory ↑GSH, AChE, BChE ↓MDA ↓THLE, GTCS	[34,35]

^1^ Unless stated in the table, such as oleamide extracted from jujube, the administered sample was the whole jujube fruit or its extract. The up arrow indicates that the indicator is This indicator is upgraded/increased/enhanced. On the contrary, the down arrow indicates that the indicator is downgraded/decreased/weakened. Notes: I/R, ischemia/reperfusion; MACo, middle cerebral artery occlusion; i.g., intragastric gavage; SOD, Superoxide dismutase; HNE, 4-Hydroxynonenal; MDA, malondialdehyde; NO, nitric oxide; BDNF, brain-derived neurotrophic factor; NBM, nucleus basalis of Meynert; p.o., per os; FRAP, ferric reducing/antioxidant power; GSH, glutathione; ACh, Acetylcholine; ALT, alanine aminotransferase; AST, aspartate aminotransferase; AChE, acetylcholinesterase; BChE, butyrylcholinesterase; GFAP, glial fibrillary acidic protein; Iba-1, ionized calcium-binding adapter molecule 1; IL-6, interleukin-1β; TNF-α, tumor necrosis factor-α; INF-β, interferon-β; i.p., intraperitoneal injections; THLE, tonic hindlimb extension; GTCS, generalized tonic–clonic seizures.

### 3.2. Prevention and Treatment of Cardiovascular Diseases

Clinical studies have reported that jujube exhibits beneficial effects on glucolipid metabolism in type 2 diabetes patients (Table 3). After 12 weeks of dietary consumption of jujube, the glycosylated hemoglobin and cholesterol content decreased significantly [36]. Meanwhile, indicators like insulin and HOMA-IR (homeostatic model assessment of insulin resistance) were significantly reduced, while QUICKI (quantitative insulin check index) and ApoA-I (apoprotein A-I) were upregulated in the patients compared with the control group, which indicated that dietary jujube improves glycemic control in diabetic patients and reduces the risk of cardiovascular diseases in humans [37]. In diabetic rats, jujube decreased serum adiponectin, thereby mitigating diabetic-induced biochemical and histopathological changes [38]. In in vitro studies, the jujube triterpene extracts (betulinic acid, oleanolic acid, and ursolic acid) promoted glucose uptake by rat L6 myotubes [22]; the jujube fruit extract reduced hyperglycemia-induced cytotoxicity of PC12 cells by inhibiting caspase 3 activation and ROS production and thus exhibited a therapeutic potential for diabetic complications [39]. Moreover, jujube has exhibited potential preventive and curative effects on other cardiovascular diseases—the cardiovascular response in L-NAME hypertensive rats was significantly attenuated by the jujube extract [40]; jujube polysaccharides attenuated oleic acid-induced lipid accumulation and increased glutamate transaminase activity in L02 cells to exerting effective hypolipidemic activity [7]; and jujube triterpenoids contributed to atherosclerosis prevention by inhibiting the formation of foam cells in human macrophages [21]. Furthermore, animal studies have shown that jujube polysaccharides alleviated insulin resistance and slowed down hyperinsulinemia development by downregulating serum insulin concentrations as well as HOMA-IR and HOMA-β [41].

### 3.3. Auxiliary Anticancer Activity

The auxiliary anticancer activity of jujube has been reported in cells (such as LoVo, MDA-MB-231, and U937 cells) and mice (Table 4). Polysaccharides and triterpenoids are potential antitumor ingredients, and the action mechanisms include the induction of apoptosis, cell cycle arrest, mitochondrial dysfunction, the reduction of inflammation, and the regulation of the intestinal microbiota. In the mice colon cancer model, dietary jujube improved the anticancer efficiency of cyclophosphamide by optimizing the intestinal microflora [45], and it reduced the number of tumors by inhibiting the activation of inflammatory signals [46]. In cell experiments, jujube polysaccharides induced G0/G1 or G2/M cycle arrest and apoptosis in colon cancer Lovo and SW620 cells, and they inhibited cancer cell proliferation [6,47]. Additionally, jujube polysaccharides exerted potential inhibitory effects on cervical and skin cancers by inducing the apoptosis of HeLa cells as well as G2/M cycle arrest and the endogenous apoptosis of melanoma cells [19,48]. The jujube triterpenoid 3-O-trans-p-coumaroyl-alphitolic acid (3OTPCA) induced the endogenous apoptosis of leukemic U937, Molt-4, and Jurkat cells by generating ROS and activating the unfolded protein response [25]. In addition, 3OTPCA induced the apoptosis of lung (A549), prostate (PC-3), and breast (MDA-MB-231) cancer cells by increasing mitochondrial ROS production and activating p38 MAPK [10].

### 3.4. Anti-Inflammatory Effects

Jujube is used as an antidote in the TCM soup “Shizao decoction” for relieving inflammatory irritation. It exerts anti-inflammatory effects mainly by affecting the growth and metabolism of inflammatory cells and cytokine release and expression (Table 5). Among the six active compound groups from jujube, triterpenic acids exhibited the strongest anti-inflammatory activity along with a significant inhibition of NO production and splenocyte proliferation [51]. According to Ruan et al. [52], among 29 triterpenoids from jujube, ursolic acid and oleanolic acid exerted stronger anti-inflammatory activity by downregulating the expression of inflammatory factors through the NF-κB signaling pathway. Animal experiments have demonstrated that jujube possesses in vivo anti-inflammatory activity, showing potential application in the treatment of inflammatory diseases such as plantar fasciitis and pneumonia. Reduced granuloma tissue formation and inflammatory mediators in rats were detected after the gavage administration of jujube extract [9]. In a dermatitis mice model, the increase in ear skin thickness and water content induced by TPA were markedly inhibited by jujube essential oil [53].

### 3.5. Hepatoprotection

Jujube is traditionally considered a tonic for enhancing liver function and preventing hepatic diseases. As shown in Table 6, in a clinical study on tuberculosis patients, jujube syrup effectively prevented antituberculosis drug-induced liver damage [58]. According to animal and cellular experiments, jujube polysaccharide and flavonoid improved liver impairment by activating the body’s antioxidant system and inhibiting inflammatory responses [8,12]. After treatment with jujube, mice with liver injury exhibited a significant recovery of liver function and an alleviation of histopathology [9]. The increased protein expression and nuclear translocation of NRF2 (a major regulator of cellular antioxidants) and the reduced NF-κB (a key transcription factor that upregulates pro-inflammatory factors) protein expression suggested that jujube protects against liver injury possibly by alleviating oxidative stress inflammation [12]. In India and China, jujube is also used for treating jaundice, a stigmata and symptom of the yellowish staining of the skin, sclera, and mucous membranes directly caused by elevated serum bilirubin levels [59]. A randomized double-blind trial revealed that the serum bilirubin levels of jaundiced newborns decreased significantly after 12 h of jujube treatment compared with those in the control group, indicating that jujube treatment is safe and effective against jaundice in the short term [60].

### 3.6. Gastrointestinal Protection

According to TCM, jujube warms and tonifies the spleen and stomach. Jujube has been traditionally used to treat gastrointestinal diseases such as anorexia and loose stools, and its paste and puree often serve to aid digestion. In some countries, jujube is utilized a symptomatic treatment for chronic constipation [62].

The protective effect of jujube on the gastrointestinal tract is reflected, on the one hand, through its ability to improve intestinal mucosal damage by altering the intestinal antioxidant status and reducing the exposure of the intestinal mucosa to harmful compounds such as toxic ammonia [63,64] (Table 7). On the other hand, jujube positively regulates the intestinal microbiota and modulates intestinal inflammatory signals and the levels of cytokines and tight junction proteins, thereby protecting the intestinal barrier function [65,66]. Moreover, jujube alleviates the symptoms of gastric ulcer, diarrhea, and constipation. Jujube stem bark extract significantly diminished the ulcer index and area, submucosal edema, and interstitial hemorrhage in mice with gastric ulcers [67].The ethanolic extract of jujube reduced the number and weight of watery stools and significantly delayed diarrhea onset in a dose-dependent manner [55]. In a clinical trial, after chronic constipation patients being treated with jujube extract drops for 11 weeks, both the objective indicators and the subjective quality of life score improved significantly, with no changes in the liver and kidney function or other adverse effects, which verified the safety and efficacy of jujube in improving chronic idiopathic constipation [62].

### 3.7. Others

In addition, jujube exerted many other pharmacological effects (Table 8). The triterpene acids, polysaccharides, and peptides in jujube exhibit substantial inhibitory effects on a few pathogenic microorganisms [16,23,68]. Jujube polysaccharides can trigger immunomodulation by stimulating immune cell proliferation and immune activity and regulating the expression of pro-inflammatory cytokines [18,69]. They also alleviated fatigue in rats with chronic fatigue syndrome [70]. The chloroform extract of jujube mediated anti-obesity effects by downregulating the expression of key adipogenic transcription factors [71]. The water extract of jujube enhanced two key processes of hematopoiesis—erythropoiesis and erythrophagocytosis—by upregulating EPO and promoting heme iron recycling, respectively [72,73]. Furthermore, jujube protected the kidney from the toxic damage induced by ibuprofen or cisplatin [24,74].

## 4. Discussion and Perspective

Jujube has been used for edible and medicinal purposes for a long time. With the growth of modern food processing and transportation industries, jujube is now consumed globally as dried fruits or preserves. As an advanced herbal medicine, it is traditionally used in the treatment of insomnia, depression, and anemia. In recent years, research on the active ingredients and medicinal effects of jujube have attracted increasing attention, which is delightful; however, it is a fly in the ointment that in many cases, jujube is confused with *Z. jujuba* var. *spinosa*, which is also known as *Z. acidojujuba* or sour jujube, and is the wild ancestor of jujube [79]. Although they both belong to the Rhamnaceae family and are in the same evolutionary chain, they differ notably in their appearance, taste, and medicinal parts and effects. For example, in TCM prescriptions, the main medicinal part of sour jujube is the seed, which is mainly used for sedative and sleeping effects. In contrast, the active part of jujube is the fruit, and it is often used for nourishing the blood and strengthening the spleen and stomach [80]. Therefore, the study of jujube and sour jujube should be carefully distinguished. This review focuses on the pharmacological effects and action mechanisms of jujube in order to provide a reference for the research and application of jujube in the medical and healthcare field.

Studies on jujube have increased significantly in the last two decades, demonstrating the diverse pharmacological activities of jujube and its efficacy against various clinical conditions, including neurological disorders and cardiovascular diseases [29,36]. However, research on jujube is still focused on the agriculture and chemical industries, whereas research on the medicinal effects, especially clinical research, remains scarce, which limits the application of jujube in clinical medicine. Therefore, the pharmacological activities of jujube should be paid more attention. Moreover, it is crucial to draw inspiration from the Chinese medical canon. For example, the medical classics Treatise on Typhoid and Miscellaneous Diseases and Shennong’s Herbal have recorded that jujube exerts mood-regulating effects such as calming the mind and improving sleep [2,4], whereas there is a paucity of research, which offers a new idea for the pharmacological research of jujube.

Jujube is rich in polysaccharides, triterpene acids, phenolic compounds, and other biologically active substances [5]. Owing to these components, jujube has many health benefits for the human body and has a great potential for development in the food and pharmaceutical industries, such as the selective development of functional foods and pharmaceuticals based on the content of specific compounds. However, most of the current studies on the pharmacological effects of jujube are conducted on the whole jujube fruit, resulting in a lack of clarity in the correspondence between the pharmacological effects of its active ingredients. Hence, the separation of the active ingredients should be carried out and then combined with advanced techniques such as genomics, proteomics, and metabolomics to unravel the active compounds and targets of jujube. Additionally, the potential pharmacological activities of jujube may be associated with the combination and content of their bioactive substances. Therefore, exploring the interactions and synergistic effects of various active components in jujube, as well as their compatibility with other natural substances or drugs, is also an important direction for future research. In addition, the active ingredients and biological activities of jujube are influenced by region, developmental stage, and other factors [81], which should be considered judiciously in the process of sample preparation. Furthermore, there is a lack of toxicological and tolerance studies on jujube, which should be taken into account to provide valuable insights into the efficacy and safety of jujube in various populations and disease conditions. Overall, a comprehensive understanding of the mechanisms and optimal utilization of jujube can pave the way for its practical applications in the medicine and healthcare fields, but further research is still needed to bridge the current knowledge gaps.

## 5. Conclusions

As a valuable medicinal material, jujube exhibits tremendous potential in the spheres of medicine and food. This review emphasizes the pharmacological effects of jujube and the corresponding active components and molecular mechanisms. In the future, researchers should place greater emphasis on the structure–activity relationship of the active components of jujube, and it is urgent to conduct toxicological research and clinical trials to verify the safety and effectiveness of jujube efficacy so that it can be better used to promote human health.

## Figures and Tables

**Table 1:** The structure and function of the active components from jujube.

Ingredients	Representative Compounds	Pharmacological Activities	Refs.
Polysaccharides	Composed of nine monosaccharides in different ratios: 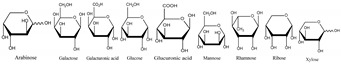	Antioxidant, anti-inflammatory, auxiliary anticancer, immune-boosting, hypolipidemic, hypoglycemic, antibacterial	[7,18,19,20]
Triterpenic acids	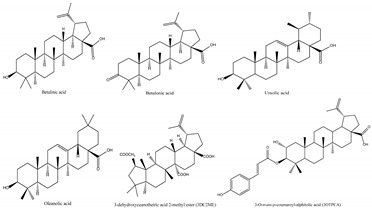	Anti-inflammatory, auxiliary anticancer, foam cell formation inhibitory, hypoglycemic, antiviral, antioxidant	[21,22,23,24,25]
Phenols	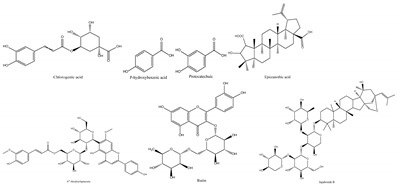	Antioxidant, anti-inflammatory, immunoregulation	[26,27,28]
Alkaloids	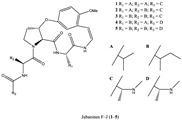	Antiviral, antioxidant	[13,14]
Polypeptides	Snakin-Z	Antibacterial, antioxidant, cholinesterase inhibitory	[16,17]
Fatty acid amide	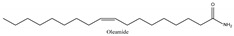	Cholinesterase activation, antineurotoxicity	[15]

**Table 3:** Therapeutic effects and potential pathways of jujube in the prevention and treatment for cardiovascular diseases.

Diseases	Models	Type	Administration	Effects	Refs.
Hyperglycemia/Diabetes	Rats: STZ	In vivo	i.g.: 0.25–2 g/kg	↓FBG, TG, HDL-C ↓ROS ↓Caspase 3 activation	[36,37,39,42,43]
	PC12 cells: glucose	In vitro	300 μg/mL		
	Patients: type 2 diabetes	Clinical	30 g/d	↑ApoA-I, QUICKI ↓FBG, TC, LDL-C, TC/HDL-C, LDL-C/HDL-C, hs-CRP, insulin, HOMA-IR, ApoB100, HbA1c ↓Percentage change of weight, BMI	
High blood pressure	Rats: L-NAME	In vivo	i.v.: 100, 200, 400 mg/kg i.g.: 150, 300 mg/kg	↓∆SBP, ∆MAP, ∆HR, tachycardia	[40,44]
High blood cholesterol	Mice: dyslipidemia	In vivo	i.g.: 200, 400 mg/kg	↑HDL-C ↓TG, ALT, TC, LDL-C, VLDL-C, hepatic steatosis, AI	[7,41]
	LO2 cells: oleic acid	In vitro	100, 200, 300 μg/mL (polysaccharides)		
Atherosclerosis	HMDM cells: Ac-LDL	In vitro	50 μM (triterpenoids)	↓Foam cells, CE, ACAT	[21]
Hyperinsulinemia	Mice: insulin resistance	In vivo	i.g.: 200, 400 mg/kg (polysaccharides)	↓Insulin, HOMA-IR, HOMA-β	[41]

Notes: STZ, sterepto-zotocin; FBG, fasting blood glucose; TG, triglyceride; HDL-C, high-density lipoprotein cholesterol; ROS, reactive oxygen species; ApoA-I, apoprotein A-I; QUICKI, quantitative insulin check index; TC, total cholesterol; LDL-C, low-density lipoprotein cholesterol; VLDL-C, very low-density lipoprotein cholesterol; hs-CRP, high-sensitivity C-reactive protein; HOMA-IR, homeostatic model assessment of insulin resistance; ApoB100, apolipoprotein B-100; HbA1c, glycosylated hemoglobin; BMI, body mass index; L-NAME, N(G)-nitro-L-arginine methyl ester; i.v., intravenous injection; ∆SBP, maximal changes of blood pressure; ∆MAP, maximal changes of mean arterial pressure; ∆HR, maximal changes of heart rate; AI, atherogenic index; Ac-LDL, acetylated lipoprotein; CE, cholesterol ester; ACAT, acyl coenzyme A: cholesterol acyltransferase; HOMA-β, homeostatic model assessment of β-cell function.

**Table 4:** Therapeutic effects and potential pathways of jujube in the auxiliary prevention and treatment for cancer.

Diseases	Models	Type	Administration	Effects	Refs.
Colon Cancer	Mice: AOM/DSS	In vivo	p.o.: 5%, 10% (*w/w*)	↑ROS ↑Apoptosis, cell cycle arrest ↓Cell proliferation ↓NF-κB/IL-6/JAK1/STAT3 ↓Leukocytes, IL1-β, TNF-α, IL-7, GM-CSF ↓Fecal blood, diarrhea, DAI, spleen weight	[6,10,46,47]
	Cells: LoVo cells, SW620 cells, HTH29 cells	In vitro	100, 200, 400 μg/mL (polysaccharides) 25–800 μg/mL (polysaccharides) 100 μg/mL		
Breast Cancer	MDA-MB-231 cells	In vitro	3, 10, 30 μM (triterpenoids)	↑Apoptosis	[10]
Leukemia	U937 cells, Molt-4 cells, Jurkat cells	In vitro	10–500 μg/mL (seed extract) 40 μM (triterpenoid)	↑Apoptosis ↑UPR, ROS ↑Caspase 3, 8, 9 activities ↑CHOP, p38 MAPK, t-BID, XBP1s, BCL2 ↓*Bcl2* ↓Cell proliferation ↓Mitochondrial membrane potential	[25,49]
Cervical Cancer	HeLa cells,	In vitro	25–400 μg/mL (polysaccharides)	↑Apoptosis ↓Cell proliferation	[19]
Lung Cancer	A549 cells	In vitro	1, 10, 50, 100 μg/mL 3, 10, 30 μM (triterpenoids)	↑Apoptosis ↓Cell proliferation	[10]
Liver Cancer	HepG2 cells	In vitro	100, 200 µg/mL	↑Apoptosis, cycle block ↑ROS ↑RB, p27^Kip1^ ↓Mitochondrial membrane potential	[50]
Skin Cancer	Melanoma cells	In vitro	2.5, 3.75, 4.25, 5 mg/mL	↑Cell cycle arrest ↑Caspase 3, 9 activity ↓Cell proliferation	[48]
Prostate Cancer	PC-3 cells	In vitro	3, 10, 30 μM (triterpenoids)	↑ROS ↑Apoptosis ↑Cleaved caspase 3, 7, 8, BID, PARP, p38 MAPK activation ↓Mitochondrial membrane potential	[10]

Notes: AOM/DSS, azoxymethane/dextran sodium sulfate; NF-κB, nuclear factor-κB; GM-CSF, granulocyte-macrophage colony-stimulating factor; DAI, disease activity index; UPR, unfolded protein response.

**Table 5:** Therapeutic effects and potential pathways of jujube in the prevention and treatment for inflammatory.

Diseases	Models	Type	Administration	Effects	Refs.
Plantar Fasciitis	Rats and mice: foot swelling	In vivo	i.g.: 200, 400 mg/kg 800, 1200, 1600 mg/kg 50, 100, 200, 400 mg/kg (root bark extract)	↓Paw oedema ↓TNF-α, IL-1β	[9,54,55]
Granulomatous inflammation	Rats: granulomas	In vivo	i.g.: 100, 200, 400 mg/kg	↓Granuloma ↓Nitrite/nitrate	[54]
Dermatitis	Mice: TPA	In vivo	1%, 10% (seed essential oil)	↓Ear thickness, water content	[53]
Pneumonia	Mice: benzo(a)pyrene	In vivo	p.o.: 1.5 g/kg 0.75 g/kg	↑NRF2, HO-1 ↑PGE2, GSH/GSSG ↓iNOS, COX-2 ↓MDA, 8-OHdG ↓lung tissue injury ↓NF-κB, TNF-α, IL-1β	[56]
	A549 cells: TPA	In vitro	500 μg/mL		
Mucositis of the oral cavity	Golden hamsters	In vivo	p.o: 300 mg/kg Application: 20%	↑MPO, SOD ↓MDA ↓Histopathology score	[57]

Notes: TPA, 12-O-tetradecanoylphorbol-13-acetate; PGE2, prostaglandin E2; GSH/GSSG, the ratio of reduced to oxidized glutathione; NRF2, nuclear factor erythroid 2-related factor 2; HO-1, heme oxygenase-1; iNOS, inducible nitric oxide synthase; COX-2, cyclooxygenase 2; 8-OHdG, 8-hydroxy-2′-deoxyguanosine; MPO, myeloperoxidase.

**Table 6:** Therapeutic effects and potential pathways of jujube in the prevention and treatment for hepatopathy.

Diseases	Model	Type	Administration	Effects	Refs.
Non-alcoholic liver injury	Mice: CCl_4_, acetaminophen	In vivo	i.g.: 100, 200, 400 mg/kg (flavonoids) p.o.: 100, 200, 400 mg/kg (polysaccharides)	↑CAT ↑NRF2 ↑SOD, GSH-Px, GSH, NQO1 ↓NF-κB ↓MDA, lipid peroxidation ↓ALT, AST, ALP, TB, LDH ↓TNF-α, IL-6, IL-1β, IL-10	[8,9,12,58]
	HepG2 cells: CCl_4_	In vitro	100 μg/mL (root bark extract)		
	Patients: tuberculosis	Clinical	10 mL/d (jujube syrup)	↑QOL ↓Cough ↓Hepatotoxicity	
Alcoholic liver disease	Mice: alcohol	In vivo	i.g.: 0.02 g/kg	↑GSH ↑Cell viability ↑NRF2, HO-1, NQO1, GCLC ↓AST, ALT ↓MDA, ROS ↓CYP2E1, TNF-α ↓Histological lesions	[61]
	HepG2 cells: alcohol	In vitro	100 μg/mL		
Jaundice	Patients: jaundiced newborns	Clinical	1 mg/kg	↓Bilirubin	[60]

Notes: CAT, catalase; GSH-Px, glutathione peroxidase; NQO1, NAD(P)H: quinone oxidoreductase 1; ALP, alkaline phosphatase; TB, total bilirubin; LDH, lactic dehydrogenase; QOL, quality of life; GCLC, glutamate-cysteine ligase; CYP2E1, cytochrome p450 2E1.

**Table 7:** Therapeutic effects and potential pathways of jujube in the prevention and treatment for gastrointestinal diseases.

Diseases	Models	Type	Administration	Effects	Refs.
Intestinal mucosal injury	Rabbits: I/R Golden hamsters	In vivo	p.o.: 200, 400 mg/kg (polysaccharides) 1.7 g, 5.0 g, 15 g/kg diet	↑SCFAs ↑Fecal moisture ↑GSH, GSH-Px, SOD, CAT, ↓MDA ↓Cecal ammonia ↓Bacterial enzymes ↓Gastrointestinal transit time	[63,64]
Intestinal barrier dysfunction	Mice: sepsis, cyclophosphamide	In vivo	i.g.: 150, 300, 600 mg/kg (polysaccharides) 200, 500, 1000 mg/kg (polysaccharides)	↑Survival ↑IgA, SIgA ↑Splenic lymphocytes ↑*Firmicutes*/*Bacteroidetes* ↑ZO-1, claudin-1, occluding ↑IL-2, IL-4, IL-10, INF-γ, TNF-α ↓DAO ↓BCL2, BAX, caspase 3 ↓TLR4/NF-κB signaling ↓CD3+ and CD4+ spleen T lymphocytes, CD4+/CD8+ ↓intestinal mucosal damage	[65,66]
Gastric ulcer	Mice: gastric ulcer	In vivo	100, 200, 400 mg/kg (stem bark extract)	↓Ulcer area, submucosal edema, interstitial hemorrhage	[67]
Diarrheal disease	Mice: acute diarrhea	In vivo	800, 1200, 1600 mg/kg	↓Diarrhea	[55]
Chronic constipation	Patients: chronic constipation	Clinical	20–40 drops	↑QOL score ↓Transit time ↓Symptom severity ratings	[62]

Notes: SCFAs, short-chain fatty acids; IgA, immunoglobulin A; SIgA, secretory immunoglobulin A; ZO-1, zonula occludens-1; DAO, diamine oxidase; TLR4, toll-like receptor 4.

**Table 8:** Other pharmacological effects and potential mechanism of jujube.

Effects	Models	Type	Administration	Findings	Refs.
Immunoregulation	Rat: chronic fatigue Syndrome	In vivo	p.o.: 100, 200, 400 mg/kg (polysaccharides)	↑IL-2 ↓IL-10 ↑CD4^+^/CD8^+^, T cell proliferation, NK cell activity	[70]
Antimicrobial	Oral pathogenic bacteria: *streptococcus mutans*, MRSA, *porphyromonas gingivalis*	In vitro	(Polysaccharides)	↓Biofilm formation, host cell adhesion, host cell invasion, cytotoxicity	[68]
Skin protection	Zebrafish larvae	In vivo	20 μM (flavonoid glycosides)	↓Melanogenesis ↓Tyrosinase activity ↓cAMP/CREB/MITF	[11]
	B16F10 cells	In vitro			
Anti-obesity	Obese adolescents	Clinical	15 g/day	↓LDL-C, TC	[75]
Hematopoiesis-enhancing	RAW 264.7 cells	In vitro	0.187–3.0 mg/mL	↑HO-1, biliverdin reductase A and B, ferroportin	[72]
Antigenotoxicity	Mice: hydroquinone	In vivo	p.o.: 0.5 g/kg (leaf extract)	↓Chromosomal aberrations	[76]
Renal protection	Rats: ibuprofen	In vivo	p.o.: 500 mg/kg	↑Albumin, total protein ↑Body weight ↓Urea, creatinine ↓Hypercellularity and shrinkage in glomeruli lines, ischemia in proximal convoluted tubules and congestion	[74]
Anti-steroidogenic	Adult female mice	In vivo	p.o.: 60, 120, 240 mg/kg (bark extract)	↓Estrus cycle ↓Wet weight of ovaries ↓Cholesterol, ascorbic acid, Δ^5^-3β-hydroxysteroid dehydrogenase, glucose-6-phosphate dehydrogenase	[77]
Anti-aging	Drosophila	In vivo	p.o.: 30, 150 mg/mL	↓lifespan, healthspan ↑d4E-BP mRNA transcript ↓mRNA levels of 14-3-3ε	[78]

Notes: cAMP, cyclic adenosine monophosphate; CREB, cAMP-response element-binding protein; MITF, microphthalmia transcription factor.

## Data Availability

Data are contained within the article and supplementary materials.

## Supplementary Materials

The following supporting information can be downloaded at: https://www.mdpi.com/article/10.3390/foods13020193/s1, File S1: The compounds structures.
